# Traffic Aware Scheduler for Time-Slotted Channel-Hopping-Based IPv6 Wireless Sensor Networks

**DOI:** 10.3390/s22176397

**Published:** 2022-08-25

**Authors:** Diana Deac, Eden Teshome, Roald Van Glabbeek, Virgil Dobrota, An Braeken, Kris Steenhaut

**Affiliations:** 1 Department of Electronics and Informatics (ETRO), Vrije Universiteit Brussel, Pleinlaan 2, 1050 Brussels, Belgium; 2 Department of Engineering Technology (INDI), Vrije Universiteit Brussel, Pleinlaan 2, 1050 Brussels, Belgium; 3 Communications Department, Technical University of Cluj-Napoca, Memorandumului 28, 400114 Cluj-Napoca, Romania; 4 Department of Electrical and Computer Engineering (ECE), Jimma Institute of Technology, Jimma University, Jimma P.O. Box 378, Ethiopia

**Keywords:** IPv6 routing protocol for low-power and lossy networks, Orchestra, time-slotted channel hopping, traffic-aware scheduling, wireless sensor networks

## Abstract

Wireless sensor networks (WSNs) are becoming increasingly prevalent in numerous fields. Industrial applications and natural-disaster-detection systems need fast and reliable data transmission, and in several cases, they need to be able to cope with changing traffic conditions. Thus, time-slotted channel hopping (TSCH) offers high reliability and efficient power management at the medium access control (MAC) level; TSCH considers two dimensions, time and frequency when allocating communication resources. However, the scheduler, which decides where in time and frequency these communication resources are allotted, is not part of the standard. Orchestra has been proposed as a scheduler which allocates the communication resources based on information collected through the IPv6 routing protocol for low-power and lossy networks (RPL). Orchestra is a very elegant solution, but does not adapt to high traffic. This research aims to build an Orchestra-based scheduler for applications with unpredictable traffic bursts. The implemented scheduler allocates resources based on traffic congestion measured for the children of the root and RPL subtree size of the same nodes. The performance analysis of the proposed scheduler shows lower latency and higher packet delivery ratio (PDR) compared to Orchestra during bursts, with negligible impact outside them.

## 1. Introduction

The increase in the number of devices connected to the Internet broadens the meaning of the latter, creating the Internet of things (IoT). Devices can be car components, household items, industrial machinery, detection systems, etc. The main objective of connecting these devices to the Internet is to exchange data. Devices that are part of the IoT can be used for sensing, actuation, remote controlling, processing, monitoring, etc. The measurements collected from these objects can aid in various domains, from healthcare, manufacturing, and agriculture to power plants and detection of unexpected natural events. The IoT devices can participate in ad hoc network formation, collaborate, collect data about the physical state of the environment, aggregate the data, and transmit them to a central processing unit. If one or multiple devices disappear from the network, the network should be able to rebuild, and communication between devices should continue unhindered as soon as possible.

The wireless sensor networks (WSNs) are a subset of the IoT world. They consist of spatially scattered and specialized autonomous devices called wireless sensor nodes. The nodes communicate among them wirelessly, often via multi-hop paths. WSNs are subjected to a combination of node and network limitations. The most important node constraints are limited energy supply, limited processing power, and limited memory. The network constraints are due to the characteristics of wireless links. They are lossy and prone to interference, and bandwidth is limited. Nodes might generate unexpected traffic load. All these constraints in combination with unpredictable traffic load fluctuations make it difficult to guarantee the desired quality of service (QoS). Protocols that govern WSNs should adapt to the resource scarcity and unpredictable character of wireless links.

The physical layer is responsible for converting the stream of bits into a form that is suitable for transmission over a wireless connection. The MAC protocol mediates or arbitrates the shared medium, and manages the radio’s power consumption. In case the nodes try to transmit at the same time, collision avoidance mechanisms are in place. Not all devices are in direct range of a central unit. Thus, a routing protocol should be able to find the best multi-hop path for communication between nodes and the central unit. In WSNs, RPL is the most common solution.

Power management implies that the node has the radio turned on when it has to send, receive or listen to the medium and has the radio turned off otherwise. One of the most promising MAC protocols is the IEEE 802.15.4e amendment published by the Institute of Electrical and Electronics Engineering Standards Association (IEEE-SA) in 2012 [1], called TSCH. For optimal functioning, TSCH needs a scheduler responsible for organizing when it is time for the nodes to switch their radio on and when it is time to switch it off.

This paper aims to develop a scheduler that allocates additional cells in case of high traffic to ensure the application’s required QoS. The scheduler is designed for convergecast traffic, and it is based on the principles of Orchestra [2]. In case of convergecast traffic, the most constrained nodes are nearest to the collecting point. For these nodes, future congestion is detected by counting the remaining packets in the queue. Additional cells are allocated for these nodes. The number of cells to be allocated is adopted considering the number of descendants that these nodes have. By considering both the congestion and the number of descendants, our solution becomes suitable also for unbalanced networks, where nodes have sizable differences in the number of descendants. For nodes with a high number of descendants, the congestion worsens in time if the traffic remains high, thus the need for more cells.

We evaluated our scheduler in the Cooja [3] simulator for two node placements for multiple traffic loads. The performance analysis shows that our solution adapts to sudden changes in traffic by allocating additional cells if there is a burst and deallocating them when the burst ends. It increases the PDR, decreases the delay, and preserves the stability of the network in case of a sudden increase in traffic. It performs similarly to Orchestra outside the burst. Allocating additional cells clearly leads to a rise in the radio duty cycle. To illustrate the strengths of our scheduler and its usefulness, we compare it with one of the state of the art approaches.

The rest of the paper is organized as follows: Section 2 describes the protocols related to the implementation of the new scheduler and discusses the Orchestra scheduler and its shortcomings. Section 3 introduces related work. Section 4 outlines the design and implementation of the subtree-size and traffic-aware scheduler. Section 5 describes the performance evaluation methods. Section 6 presents the results, and Section 7 concludes the paper.

## 2. Background

### 2.1. RPL

**Figure 1:**
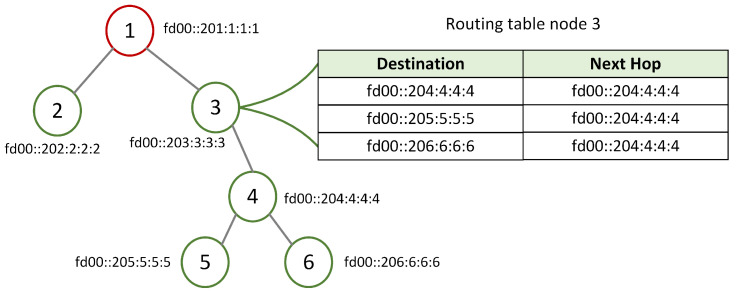
Example of routing table created by the IPv6 routing protocol for low-power and lossy networks (RPL) in storing mode for node 3.

As already mentioned, this research proposes a scheduler for TSCH-based WSNs that can allocate additional cells during traffic bursts. The additional number of cells is computed based on the subtree size. This latter information can be obtained from the routing protocol. The de facto routing protocol in WSNs, RPL [4], is optimized for convergecast traffic and was proposed by the Internet Engineering Task Force (IEFT). RPL organizes the network in a destination-oriented directed acyclic graph (DODAG) with the help of control messages. The DODAG information object (DIO) message is sent from the root to the nodes in the network, and it advertises the existence of the RPL tree and its properties. The nodes that receive this message compile a list of candidate parents with the help of an objective function and choose a preferred parent from the list. The subtree of a node consists of the children of the node and all the descendants of the children. Since we are interested in the size of the subtree, RPL is set to work in storing mode. In this case, as a response to the received DIO message, the nodes send a destination advertisement object (DAO) message to their preferred parent. This DAO message is forwarded upward by the ancestors until it reaches the root. Based on the information in the DAO message, each node can keep a routing table with destination and next hop entries for all the nodes in its subtree, as shown in Figure 1. The subtree size of a node is the number of entries in the routing table of that node. In Figure 1, the subtree size of node 3 is 3 (nodes 4, 5 and 6 form the subtree). Once nodes are part of the tree, they also broadcast the DIO messages. Nodes might request to join the network before receiving the DIO message by broadcasting a DODAG information solicitation (DIS) message.

The DIO messages are issued periodically with the help of the trickle timer algorithm. The role of the trickle timer is to tune the frequency at which control messages are sent in a network. If inconsistencies in the network are observed or if the connectivity changes, the rate at which DIO messages are sent is increased [5].

### 2.2. TSCH

Frequently used MAC methods are time division multiple access (TDMA), random access protocols, or a combination of both. One of the most used random access protocols is carrier sense multiple access (CSMA). Nodes listen to the channel before transmitting. The back-off mechanism of CSMA is used in case more than one node tries to transmit at the same time. In TDMA, the nodes access a medium only in their allotted cell. Clocks of devices drift, and thus nodes need to periodically synchronize.

TSCH is gaining popularity as a time-slotted MAC layer protocol. TSCH introduces multi-channel access to increase bandwidth, and channel hopping mechanism to tackle external interference and multipath effects. TSCH is responsible for granting access to the medium. It does so by organizing the medium in cells that are grouped in slotframes. These slotframes repeat periodically. A slotframe cell has as coordinates the time offset and channel offset. The absolute slot number (ASN) counts how many cells have passed since the start of the network, and a node uses it, together with the channel offset, for channel selection [6]. TSCH uses special control packets called enhanced beacons (EBs) to ensure that each node has its clock synchronized to the time source.

### 2.3. Orchestra

Orchestra [2] is an autonomous scheduler for RPL-based TSCH networks. This means that each node in the network computes its schedule based on information collected from RPL, without any additional communication overhead. The resulting schedule of a node is composed of different slotframes, each dedicated to a specific traffic type: one for TSCH EBs, one for RPL control traffic and broadcast traffic, and one for unicast data traffic.

**Figure 2:**
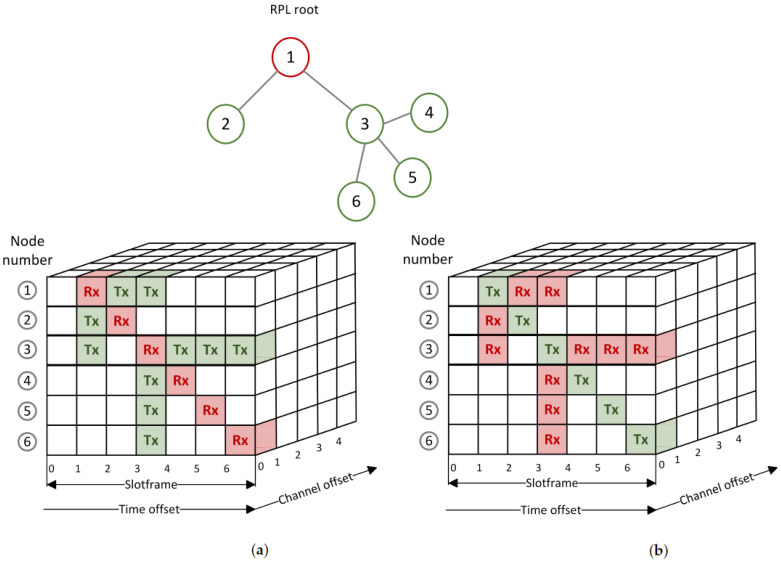
Example of scheduled Rx and Tx cells for nodes 1 to 6 in the time/channel offset space. The Rx cells are highlighted with red and the Tx cells with green. (**a**) Receiver-based shared (RBS) cell allocation. (**b**) Sender-based shared (SBS) cell allocation.

The transmission and reception cells are allocated with the help of rules. There are four types of cells: common shared (CS), receiver-based shared (RBS), sender-based shared (SBS), and sender-based dedicated (SBD). For CS cells, there is one allocated cell in the slotframe to be used by all the nodes in the network. CS cells are typically used for RPL control messages. In the case of RBS cells, one receive cell and as many transmit cells as the number of RPL neighbors are allocated. Both the time offset and channel offset are computed based on the address of the receiving node with the help of a hashing function. The role of the hashing function is to reduce the chances of a collision, which can occur when two inputs, in our case link layer addresses of nodes, result in the same value, time slot and channel offset. In the case of SBS cells, the time slot and channel offset are computed based on the parameters of the transmitting node. For each node, one cell is allocated for transmitting, and as many cells as the number of RPL neighbors for receiving. If the number of nodes in the network is lower than the number of cells in a slotframe, it is possible to use SBD cells [2]. The allocation of the cells for RBS and SBS Orchestra is visualized in Figure 2.

Some of the immediate disadvantages in the case of RBS cells are contention, and an increase in delay for bursty traffic. Moreover, in the case of both upstream and downstream traffic, the children contend with their parent for cells. Considering node 3 in Figure 2a, one can observe that there is only one receive cell for all its children. If at least two children send messages to their parent simultaneously, they compete for the same receive cell. The back-off mechanism of CSMA is used to try to avoid future collisions. This leads to an increase in delay. The more children a node has, the more severe the problem can become. In the case of SBS cells, a node has only one allocated cell per slotframe for transmitting packets (Figure 2b node 3). The packets received from its children are queued until they can be forwarded. In this situation, the latency increases, and depending on the traffic load, the queues might overflow. These drawbacks appear when traffic load increases above a certain value.

## 3. Related Work

**Figure 3:**
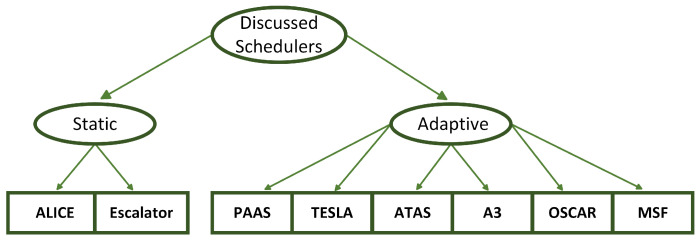
Overview of discussed schedulers.

The related work is split into static approaches and adaptive approaches. We consider the schedulers that take traffic load into consideration when allocating resources to be adaptive approaches. In contrast, the schedulers that do not consider traffic load are considered static approaches. The discussed approaches are shown in Figure 3.

### 3.1. Static Approaches

In this subsection, two autonomous schedulers are described. The first one is based on Orchestra, and it improves some of the shortcomings already discussed in Section 2. The second approach is mainly designed for upward traffic.

#### 3.1.1. ALICE

The autonomous link-based cell scheduling for TSCH (ALICE) [7] is one of the first schedulers that improves the performance of Orchestra. It keeps the structure of the slotframes dedicated to EBs and RPL control traffic but changes the structure of the slotframe dedicated to unicast traffic. For the latter, the authors of ALICE imagined a link-based approach. Instead of allocating one cell per node, the scheduler allocates one cell for each direction of communication between nodes. This way, the scheduler separates upstream and downstream traffic and avoids collisions. Additionally, the scheduling of cells changes in time to avoid repeating possible overlaps in the time slot allocation between the same nodes. The overlaps could appear because of the hashing function yielding the same cell in the slotframe to send or receive on. The authors break the repetition by introducing a new variable, the absolute slotframe number (ASFN), to compute the time offset. Other improvements to Orchestra are the early drop of packets and the impromptu matching between the packet and cell. The early drop of packets is achieved by checking if the packets’ MAC address in the queue corresponds to one of the existing neighbors’ addresses. The impromptu matching between cell and packet is performed by allocating the communication cell at the moment the packet is ready to be sent and not when the packet is added to the queue, which is the case for Orchestra. ALICE, implemented in Contiki, is compared to Orchestra, demonstrating its superiority in latency, reliability, and energy consumption. One of the disadvantages of ALICE is that it does not adapt its schedule to traffic changes.

#### 3.1.2. Escalator

Escalator [8] uses two slotframes to deal with the traffic. One slotframe is for RPL control traffic and downward traffic (the baseline slotframe) and the other is for TSCH traffic and upward traffic (the convergecast slotframe). On the convergecast slotframe, Escalator schedules one transmit cell for every node, based on the node’s parameters. This cell is used to transmit data originating from the node to its parent. For all the nodes in the subgraph of a node, two additional cells are allocated: one for receiving packets and one for transmitting them to the parent. In this case, the timeslot offset is computed based on the parameters of the nodes in the subgraph. This implementation reduces the end-to-end delay and increases PDR. Compared with Orchestra, Escalator performs better in dense networks with high traffic loads. The drawback is an increase in the average duty cycle, even for low traffic.

The discussed schedulers tackle several drawbacks of Orchestra, but they do not adapt to traffic bursts. Our scheduler, described in Section 4, adjusts the schedule in case of traffic bursts. During high traffic loads the most congested nodes are the children of the root. Congestion is measured at the level of these nodes by counting the remaining packets in their queues.

### 3.2. Adaptive Approaches

The adaptive approaches are designed for applications where the traffic load tends to create congestion. In all discussed approaches, additional resources are allocated to cope with the traffic increase. This obviously leads to an increase in the duty cycle.

#### 3.2.1. PAAS

The parameterized slot scheduling for adaptive and autonomous TSCH networks (PAAS) [9] is a solution that finds a compromise between RBS and SBS Orchestra. The implementation is based on SBS Orchestra, and in the initialization phase, it allocates for each node one Tx cell and a number of Rx cells equal to the number of children. If the measured traffic load is lower than the computed threshold, nodes use the same cell to listen to multiple children, reducing the number of Rx cells. The information needed to adapt the schedule is sent via RPL DIO messages. This scheduler maintains the same reliability and delay performance as SBS Orchestra but lowers energy consumption. The PAAS scheduler ensures better reliability and delay performance than RBS Orchestra, but leads to higher energy consumption. Its shortcoming is that it allocates first the Tx cell which might lead to lost packets if the node starts to transmit before the corresponding Rx cell is allotted.

#### 3.2.2. TESLA

The traffic-aware elastic slotframe adjustment scheduler (TESLA) [10] follows the same allocation of slotframes as Orchestra, except for the unicast slotframe. Instead of one slotframe, where both receive and transmit slots are allocated, TESLA proposes two slotframes, one for receiving unicast traffic and another for transmitting unicast traffic. Each node adjusts the size of the receiving slotframe to the traffic load. The traffic load information is piggybacked on the packets received from neighbors. If the size of the receiving slotframe changes, the node informs its neighbors about the change so they can adapt their transmitting slotframe size. The TESLA scheduler is compared on testbeds with TSCH minimal scheduling, RBS and SBS Orchestra. TESLA performs better with respect to reliability and energy efficiency but introduces overhead whenever the receiving slotframe size changes.

#### 3.2.3. ATAS

The authors of [11] propose an autonomous traffic-aware scheduler (ATAS). Through their approach, cells are allocated dynamically based on the traffic load. If the number of remaining packets in the queue is high enough, N consecutive transmitting cells are allocated. N is computed based on the size of the slotframe. Since transmit and receive cells should be synchronized, the value of N is sent in the footer of the next packet. The node that receives the packet adjusts the number of receive cells accordingly. In this approach, the traffic is also differentiated based on priority by adding the low priority packets at the tail of the queue and the high priority packets at the head of the queue. The authors evaluate their implementation in simulation and in real, based on three performance metrics: the average end-to-end delay, the average PDR, and the average duty cycle. Compared to RBS Orchestra, this approach improves the end-to-end delay and prioritizes the critical traffic.

#### 3.2.4. $A^{3}$

The scheduler proposed in [12], $A^{3}$, is fully autonomous and adapts to the traffic load. The sender predicts the traffic load by counting the remaining number of packets in its queue. This number is compared to a threshold, and cells are allocated consequently. The receiver estimates the incoming traffic by sampling the channel status without any additional information from the sender. The scheduler is implemented in Contiki and is evaluated on an open-source testbed, from which it is concluded that it improves PDR and delay compared to RBS Orchestra and ALICE for high traffic loads.

#### 3.2.5. OSCAR

The scheduler proposed in [13], optimized scheduling cell allocation algorithm for convergecast (OSCAR), exploits the principles of Orchestra. The design of the scheduler is based on the assumption that for convergecast traffic, the nodes closer to the root have higher traffic loads. The node’s rank is used to attribute a class to each node, and nodes closer to the root receive additional cells. Allocated cells are reduced if the traffic load decreases but are re-allocated if the traffic load increases again. The authors compare OSCAR with RBS Orchestra and, in the evaluated scenarios, OSCAR demonstrates to be the superior scheduler in terms of average latency and average PDR.

#### 3.2.6. MSF

The minimal scheduling function (MSF) [14] is designed to work on top of the 6TiSCH operation sublayer (6top) protocol [15]. It allocates two types of cells, autonomous and negotiated. The autonomous cells, one Rx and one Tx cell, are allocated to ensure the minimum amount of resources for bidirectional communication between neighbors. The Tx-negotiated cells are added when bursts of traffic are detected in the network. The negotiation implies the exchange of messages between the nodes, making this solution a distributed one.

### 3.3. Discussion

An overview of all the discussed schedulers is presented in Table 1.

Alice and Escalator are static approaches, meaning that the nodes allocate the same number of cells regardless of the traffic requirements. Not adapting to traffic can lead to packet loss, increased latency, and network instability. In contrast, our scheduler exploits an adaptive approach. Like in OSCAR, its design principles are based on the assumption that, in case of convergecast traffic, the nodes that suffer most are the children of the root. In low traffic conditions, OSCAR and our scheduler behave like RBS Orchestra. The traffic load is measured for the children of the root, and additional cells are allocated if it is high. Instead of using the rank to compute the number of needed additional cells, as done in OSCAR, we consider the subtree size. For unbalanced networks, in which the children of the root might have a significant difference in number of descendants, the subtree size is a relevant parameter for determining how much loaded the children of the root can get in case of high traffic. The problem of unbalanced networks is discussed in [16]. In our solution, the subtree size, which indicates how many cells should be added, is passed between nodes using an existing field of the DAO message. A similar approach is used in PAAS. The latter sends the information needed to adapt the schedule via DIO messages. Instead of using an existing field, the authors add another field to the DIO message, increasing the control traffic overhead. Moreover, in PAAS, Tx cells are allocated before Rx cells, which might lead to packet loss if the nodes start sending messages on Tx cells that do not have a corresponding Rx cell on the receiver side. We tackled this problem by waiting for the Rx cells to be allocated before adding the Tx cells.

The subtree size of the root’s children indicates how many additional cells should be allocated but not when. The root’s children monitor their queues to anticipate congestion and adapt their schedule. Additional cells are allocated if the number of remaining packets exceeds a threshold, denoted activation_threshold. If the number of remaining packets in the queue drops below the deactivation_threshold, the additional cells are removed, and our scheduler will again behave like RBS Orchestra. The authors of ATAS also determine congestion by looking at the remaining packets in the queue. The nodes react and allocate consecutively a number of cells equal to the number of remaining packets in the queue. The congestion is dealt with immediately, but allocating cells consecutively might lead to unwanted collision with the schedule of the neighboring nodes.

The receiver node needs to allocate/deallocate the corresponding communication cells. In our implementation, a flag for activating/deactivating the scheduler is piggybacked on the next unicast data packets. At this point, our approach is not fully autonomous anymore. Still, it ensures a higher average PDR, lower average delays, higher stability, and lower-to-no dropped packets from the queue without creating overhead. In other adaptive schedulers, such as TESLA and MSF, overhead is created each time the nodes need to adapt their schedule. An increase in control traffic overhead not only hinders the transmission of data packets but also leads to an increase in duty cycle. The $A^{3}$ scheduler avoids these problems by using estimation techniques for matching the number of cells that should be added on both the receiver and sender sides. This approach increases the overhead of CPU because of the computational complexity of the estimation techniques.

In all the discussed adaptive approaches, the additional resources are deallocated once they are not needed anymore.

## 4. Design of the Subtree-Size and Traffic Aware Scheduler

Orchestra is an elegant autonomous scheduler that organizes the communication resources for TSCH-based networks without any overhead or central unit. The information needed for allocating the cells is obtained from RPL. However, as already discussed in Section 2.3, Orchestra has its shortcomings, mainly in high traffic load situations. Packets might become lost due to queue overflow, or delays might increase due to congestion. In the case of high upward traffic load, the nodes that suffer the most are the root’s children. All the traffic destined for the root passes through these nodes. The proposed scheduler improves the behavior of Orchestra in high traffic load situations by allocating more transmission opportunities for the children of the root, and consequently more Rx cells for the root.

If traffic bursts are not detected by the children of the root, the cells on the unicast traffic slotframe are allocated as in RBS Orchestra: one Rx cell for each node and as many Tx cells as the number of RPL neighbors. If the children of the root detect high traffic loads, additional cells are allocated for these nodes and the root. A combination between two parameters is used to determine how many additional cells should be allocated and when will these cells be allotted. The number of remaining packets in the queue is used to predict the increase in traffic, thus congestion. The subtree size of the root’s children determines how many additional cells are needed for each child. Information about the subtree size can be collected when RPL is in storing mode. In this case, each node keeps a routing table with entries for all its descendants. The number of entries indicates the subtree size.

The slotframes dedicated to EBs and RPL control traffic have the same structure and follow the same rules as in Orchestra. The proposed scheduler contains a new rule implemented for the unicast data slotframe. This rule has the same design as all the other rules that govern the functioning of Orchestra with some additions. In each rule, including the new one, specific functions are called when a node selects or changes its parent, when a child is added/removed, or when a packet is ready to be sent. In our implementation, specific functions are also called when a packet is received and when a packet is sent. Whenever a packet is received, a function that determines the subtree size of each root’s child is triggered together with another function that decides if adding/removing more cells is necessary. This is done by checking the state of a flag previously inserted in the data packets. Whenever a packet is sent, congestion is checked by counting the remaining packets in the queue. Additional design and implementation details are discussed in the following paragraphs. Note that the description of how cells are allocated refers only to the unicast slotframe.

### 4.1. Initialization Phase

**Figure 4:**
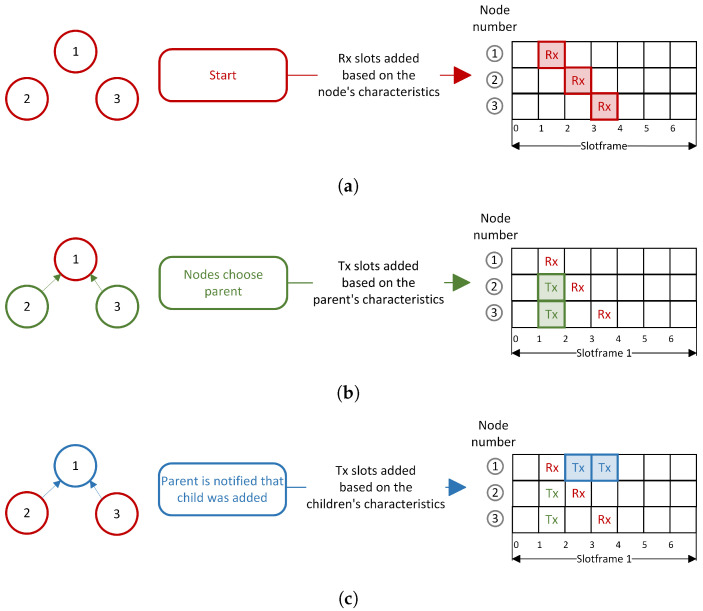
Initialization phase: allocation of Rx and Tx slots. (**a**) Initialization phase step 1: Rx slots are allocated for each node. (**b**) Initialization phase step 2: Tx slots are allocated for children to communicate with parent. (**c**) Initialization phase step 3: Tx slots are allocated for the parent to communicate with children.

This phase overlaps with the building of the RPL DODAG. The steps of the initialization phase can be visualized in Figure 4. At the start of the network’s formation, each node is allocated one Rx cell. The time slot offset is computed based on the node’s characteristics with the help of a hashing function (Figure 4a). When nodes choose a new parent, one Tx cell is allocated, with the time slot offset computed based on the parent’s characteristics (Figure 4b). Nodes are informed when a new child is added, and a Tx cell is allocated, with the time slot offset computed based on the child’s characteristics (Figure 4c). In all steps, the channel offset is computed based on the characteristics of the node. When nodes send messages, a new channel offset is computed, now based on the characteristics of the receiver. Up to this point, the functioning of our scheduler is identical to RBS Orchestra.

In our implementation, the root node keeps track of the subtree size of all its children. As a reminder, the root’s children subtree size is further used to determine the number of additional cells allocated for each child of the root. Knowing the children’s subtree sizes is not immediate. Since RPL is in storing mode, we can use the DAO messages to piggyback the subtree size. DAO messages are sent in response to DIO messages whenever a child chooses a parent. In the storing mode, DAO messages are sent to the parent. Upon receiving a DAO message, the parent adds a new entry in the routing table with information about the new node. The DAO message is forwarded to the parent’s ancestors, including the root. Whenever the root receives DAO messages from its children, it extracts and stores the subtree size information. The *Reserved* field in the DAO message is used to piggyback the subtree size. This field is normally set to zero and ignored by the receiver. By changing this specific field, we do not hamper the flow of the RPL control messages exchange and the building of the DODAG.

**Figure 5:**
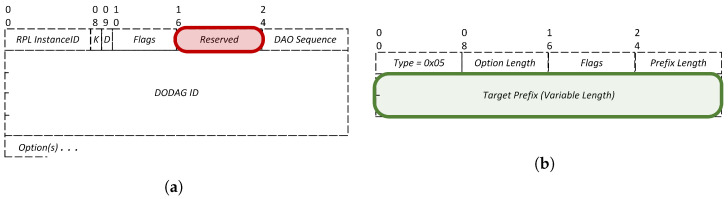
The structure of the DAO message and the RPL target option. The fields used in our implementation are marked. (**a**) Destination advertisement object (DAO) message structure. The *reserved* field is used for inserting the subtree size. (**b**) The structure of the RPL target option.

The root node receives through the DAO messages information about all the nodes in the network. We are only interested in the subtree size of the children of the root, and therefore we check the *target prefix* field in the RPL target option of the DAO message to know to which node the information belongs to. Figure 5 illustrates the structure of a DAO message (Figure 5a) and the RPL target option (Figure 5b).

At the end of this phase, the RPL tree is formed, cells for unicast traffic are allocated for all nodes in the network based on the principles of RBS Orchestra, and the root node keeps a list of all its children and their subtree size. By using the DAO message to inform the root about the subtree size of its children, we ensure that the information is kept up to date. Whenever there is a change in the network DAO messages are sent upward to inform the parents and ancestors about the changes.

### 4.2. Additional Resource Allocation Phase

The additional cells are allocated only in case of traffic burst. Upcoming congestion is measured by the root’s children by counting the number of remaining packets in the queue whenever they send a packet. If this number exceeds a threshold, denoted activation_threshold, five times, the nodes inform the root that congestion is a threat, by setting the activation flag to true. The activation_threshold is chosen based on analysis of simulations of RBS Orchestra in different traffic situations. We concluded that the average delay starts increasing considerably, and the average PDR starts decreasing when the traffic rate is higher than 5 packets per minute. In this scenario, the average number of remaining packets in the queue for the root’s children is 5. Thus, we chose 5 as an activation_threshold.

After being set to true, the activation flag is inserted in the unicast data packets destined for the root. On receiving unicast data packets, from a child, with the activation flag set to true, the root adds more Rx cells for that child. The number of additional cells is correlated with the subtree size that the root previously stored. There is a delay of 1 s between when the root adds Rx cells and when the child adds the corresponding Tx cells. The delay avoids adding Tx cells and possibly sending packets before the root can receive on the corresponding cell and is computed considering the time slot duration and slotframe size plus a safety margin. If the subtree size is less than a subtree_lower_threshold, one additional cell is allotted. If the subtree size is higher than a subtree_upper_threshold, N cells are added. If the subtree size is in between the two thresholds, M cells are added. The subtree_lower_threshold and subtree_upper_threshold are set to 3 and 5. These two thresholds are computed based on the queue lengths and based on the maximum number of routes allowed in the routing table. M and N are directly related to the slotframe size and the maximum number of children the root can have. We kept the default sizes for all the slotframes. For the unicast slotframe, the slotframe size is 17. In the scenarios that will be used to evaluate the scheduler, the root does not have more than 4 children. Accordingly, M equals 2, and N equals 3. All thresholds can be adjusted based on the specifics of the application, slotframe size, and queue lengths.

**Figure 6:**
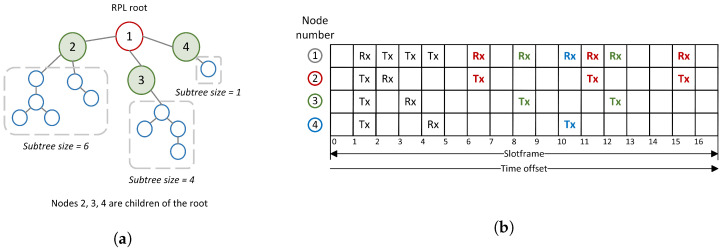
Example of allocation of Rx and Tx slots with the new scheduler. (**a**) The placement of nodes results in a topology with 3 children for the root. Each child has a different subtree size. (**b**) Allocation of Tx and Rx slots based on the topology in (**a**).

The time slot offset of the additional cells is computed using a hashing function based on the characteristics of both the root and child. Figure 6b illustrates an example of where additional slots could be added in case the children of the root have the subtree sizes shown in Figure 6a. Table 2 shows the values of all the thresholds used to build the schedule in Figure 6.

### 4.3. Deallocation Phase

The cells are deallocated when the number of remaining packets in the queue is below the deallocation_threshold for five consecutive times. Based on analysis of simulations of RBS Orchestra in different traffic situations, we concluded that, when the traffic rate is 1 or 2 packets per minute, the number of remaining packets in the queues for the root’s children rarely reaches 2 packets. Thus, the deactivation_threshold is set to 2. When the condition for deallocating cells is met, the activation flag is set to false and inserted in the following unicast packets. Upon receiving a unicast packet from one of its children with the activation flag set to false, the root removes the additional Rx cells. In the deallocation phase, the Tx cells are removed first, followed by the Rx cells, to avoid sending packets in a cell that does not have a corresponding receive cell on the root’s side.

**Figure 7:**
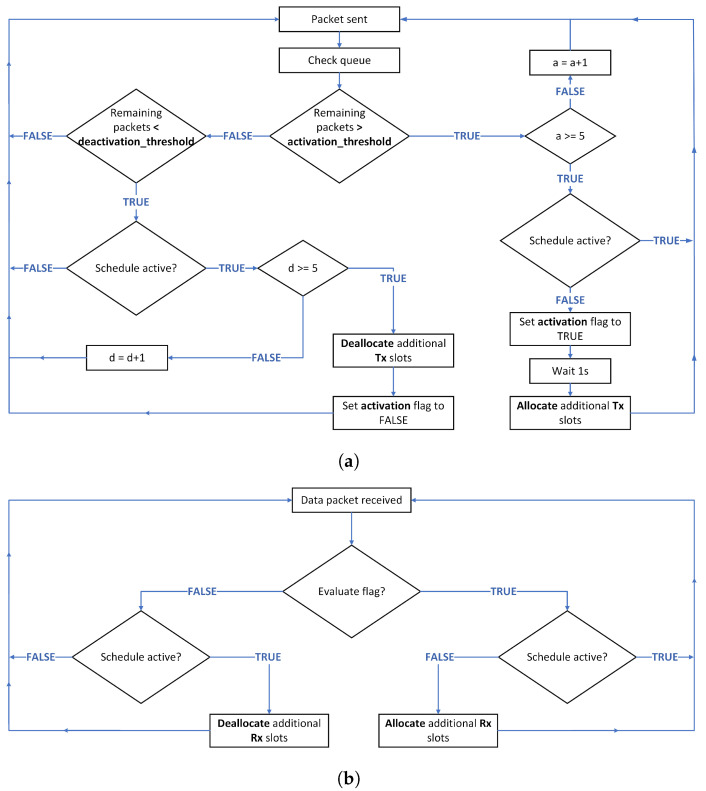
Summary of the allocation and deallocation phases for the root and its children. (**a**) The algorithm for allocating and deallocating Tx slots for the root’s children. (**b**) The algorithm for allocating and deallocating Rx slots for the root.

Figure 7 summarizes the allocation and deallocation phases. The algorithm for allocating and deallocating Tx cells for the root’s children is presented in Figure 7a. Whenever a packet is sent, each root child checks its queue. If the number of remaining packets is above the activation_threshold five times (denoted “a” in Figure 7a) and the new schedule is not already activated, the corresponding number of Tx cell is added after 1s. We check the activation condition “a” times to avoid reacting too soon and increasing the duty cycle in case the high number of packets is related to control traffic or a burst with a duration shorter than the reaction time of our algorithm. The same reasoning is applied when deallocating Tx cells, with the difference that we do not wait before removing the Tx cells.

In Figure 7b, the algorithm for allocating and deallocating Rx cells for the root is described. Whenever a data packet is received, we evaluate the activation flag. If it is true and the schedule is not already activated, we proceed with allocating Rx cells. If the activation flag is false and the schedule is active, we deallocate the Rx cells.

## 5. Materials and Methods

### 5.1. Evaluation Environment and Placement of Nodes

**Figure 8:**
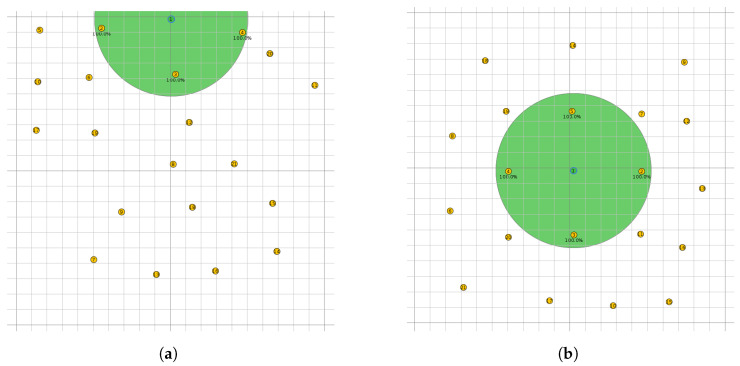
Possible placements of nodes. The green area represents the transmission and interference ranges, both equal to 50 m. The size of the grid is 200 m × 200 m. (**a**) Placement 1: the sink node is situated at the top of the grid. (**b**) Placement 2: the sink node is situated in the middle of the grid.

The proposed scheduler is implemented in Contiki-NG [17] and is evaluated in the Cooja simulator [3]. Two placements of nodes, illustrated in Figure 8, are considered. In the first one, Figure 8a, the sink node is placed at the top of the grid, and the sending nodes are positioned below. This placement of nodes facilitates the creation of a RPL topology where the children of the root have different subtree sizes. In the second placement, Figure 8b, the sink node is situated in the middle of the grid and the sending nodes around. This placement of nodes results in a RPL topology in which the root’s children have a similar subtree size. A total of 21 nodes (1 sink and 20 sender nodes) were used in all placements. The transmission and interference ranges are equal (50 m) and are identical for all the nodes. The size of the grid is 200 m × 200 m.

### 5.2. Traffic Load

The goal of the studied simulations is to analyze how the new scheduler behaves compared to Orchestra RBS in a situation where there is a sudden increase in traffic. All 20 nodes send messages toward the root after the RPL tree is formed and the network is considered to be stable. Initially, each node issues messages at a low rate (1 packet per minute and 2 packets per minute). Jitter is added in between messages to mimic the behavior of a real network. After 250 messages, the rate is increased (5, 10, 15 packets per minute). At these high rates, each node sends 500 messages. As a final step, the traffic load is decreased again to 1 or 2 packets per minute and 250 messages are sent by each node, with the exception of the sink. The figure in the Table 3 shows a visual representation of the traffic in the network.

In Table 3, all the parameters of the evaluation environment and the placements of nodes are given. Simulations are run 10 times for each scenario. Confidence intervals are computed and are indicated with the measurements. The confidence level is 95%.

### 5.3. Performance Parameters

As QoS parameters, we choose the delay, PDR, duty cycle, number of parent switches, and number of packets dropped from the queue.

The **delay** is measured at the application layer. The **average delay** is the average of the delay measured for all unicast data packets over all simulation runs related to a given scenario.

The **PDR** is the total number of unicast data packets received at the application layer of the receiver (the root) divided by the number of unicast data packets sent from the application layer of all packet sources. The **average PDR** is the average of the PDR over all simulation runs related to a given scenario.

The **number of parent switches** is the number of times each node changes parent during the simulation run. By averaging the values obtained for all sender nodes in the network across all the simulations for a given scenario, we obtain the **average number of parent switches** during a simulation run. In Contiki-NG, logging can be activated. In this case, each node prints helpful information in the output. The number of parent switches is retrieved from the nodes’ output by filtering relevant keywords and counting the number of times they appear in the course of the simulation run. Frequent parent switches will result in frequent route modifications, making RPL operate in an unstable manner. Reconstructing the routes generates additional control traffic, which uses energy and shortens the network lifetime [18]. Thus, we can consider the average number of parent switches as a performance parameter to assess network’s stability.

Conclusions regarding the average power consumption of a node can be drawn based on the **radio duty cycle**, which estimates the fraction of time the radio of a node is on in a defined period. The **average radio duty cycle** is obtained by averaging the radio duty cycle of all nodes in the network across all the simulations for a given scenario.

The **number of packets dropped from the queue** refers to all the messages dropped regardless if they are control packets or data packets. By averaging the dropped packets of all sender nodes in the network, across all the simulations for a given scenario, we obtain the **average number of dropped packets from the queue**.

The QoS parameters are analyzed during regular traffic and burst traffic.

## 6. Discussion of Simulation Results

This section analyzes the behavior of RBS Orchestra and our scheduler under different traffic loads. The two placements of nodes described in Section 5 are used for the performance analysis. The evaluation environment and the new scheduler parameters are as described in Table 3. We study the reliability of the network, average delay, and the stability of the network when there is a sudden increase in traffic. RBS Orchestra is a static scheduler. Thus, the allocated resources do not adapt to a sudden increase in traffic. Our approach allocates additional resources if the root’s children detect a potential collision. Considering these, we expect our approach to perform better than RBS Orchestra during traffic bursts and perform equally well during low traffic. We also analyze the impact the number of nodes has on the performance of the new scheduler and compare it with a state of the art adaptive approach.

### 6.1. Impact of Traffic Load and Placement of Nodes

For a fair comparison and a more profound understanding, it is essential to study both the impact of the traffic load and placement of nodes. The Section 6 graphs use placement 1 to refer to the scenario where the sink node is placed at the top of the grid, and placement 2 to refer to the scenario where the sink node is placed in the center of the grid.

#### 6.1.1. Average PDR and Number of Packets Dropped from the Queue

**Figure 9:**
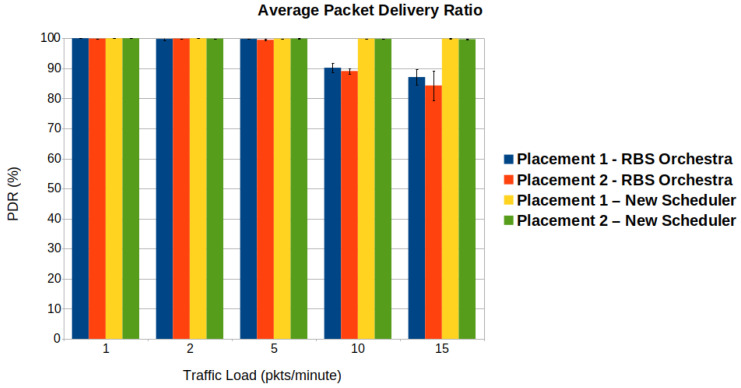
We observe that when it comes to average packet delivery ratio (PDR), the new scheduler performs better than Orchestra, in high traffic situations.

To analyze how reliable the schedulers are, we consider the average PDR, and the average number of packets dropped from the queues. Figure 9 illustrates the average PDR in the function of the traffic load. One can observe that both schedulers obtain similar performance for traffic loads lower than 10 packets per minute. For the new scheduler, the average PDR remains above 95%, regardless of the traffic load. This is a consequence of adding more cells for the most constrained nodes, namely the root’s children. Another important observation is that for placement 2, the PDR is slightly lower, especially for RBS Orchestra. This phenomena is mostly visible when the traffic is above 5 packets per minute. In placement 2, the nodes are situated in an almost circular way around the sink node. The nodes have more neighbors in their vicinity, hence there is more interference.

**Figure 10:**
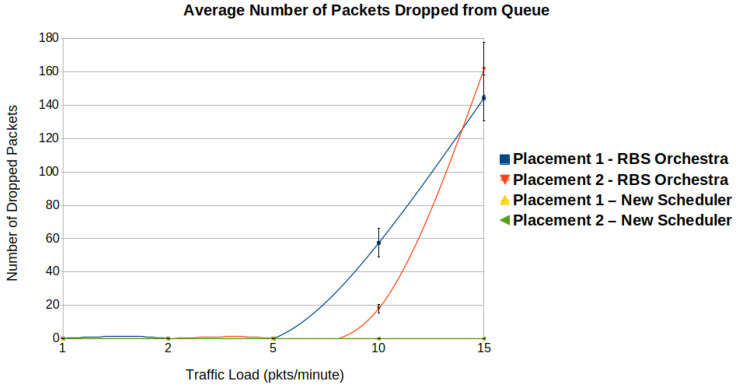
We observe that when it comes to average number of packets dropped from queues, the new scheduler performs better than Orchestra, in high traffic situation.

Figure 10 shows the average number of packets dropped from the queue in function of traffic load.

One can see a significant increase in dropped packets for RBS Orchestra when the traffic is above 5 packets per minute. The nodes that often drop packets are the root’s children. These nodes must pass the traffic from their entire subtree to the root, using only one Tx cell. Placement 1 has the root node situated at the top of the grid. The sender nodes are placed below and form denser subtrees. This explains why for placement 1, the number of packets dropped is higher. There are few to no dropped packets for the new scheduler.

#### 6.1.2. Average Delay

**Figure 11:**
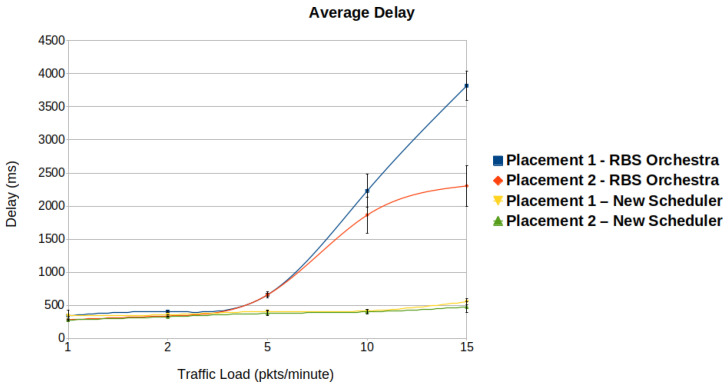
We observe that when it comes to average delay, the new scheduler performs better than Orchestra, in high traffic situation.

The delay in function of traffic load is illustrated in Figure 11. It can be immediately observed that the new scheduler maintains a low delay even for high traffic loads. When the traffic load increases above 5 packets per minute, RBS Orchestra cannot cope anymore with the high number of packets that needs to be forwarded toward the root and received by it. The reason behind this is that only one Rx cell exists at the root for all its children. For both schedulers, placement 2 performs better than placement 1. One of the reasons is that in placement 1, the number of hops between the leaf nodes and the root is higher than in placement 2.

#### 6.1.3. Average Delay and PDR Illustrated for Each Node

Showing the average delay and PDR for each node can better illustrate the performance of our scheduler and the fact that the nodes suffering the most from congestion are the children of the root. For the simulation runs, we use placement 2 (Figure 8b). During the burst, the traffic load is set to 10 pkts/minute. Before and after the burst, the traffic is set to 1 pkt/minute.

**Figure 12:**
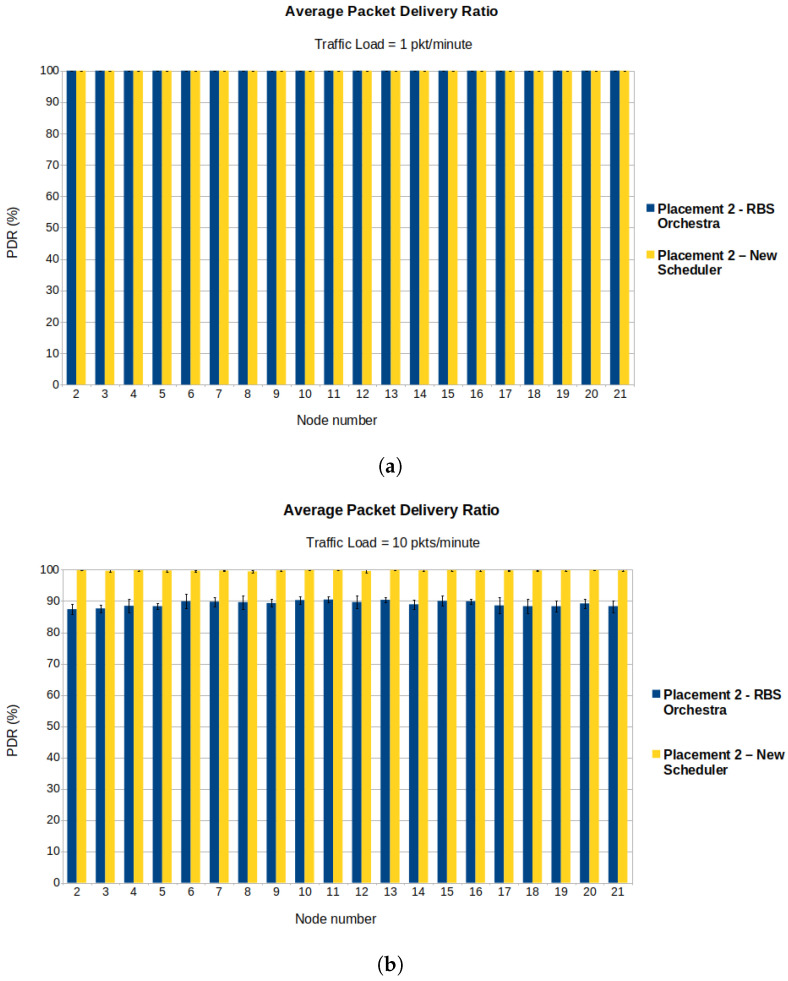
Average PDR for each sender node in low and high traffic situations. (**a**) We observe that in normal traffic situation, the average PDR is high for both schedulers. (**b**) We observe that in a high traffic situation for RBS Orchestra, the average PDR is lowest for the root’s children, being nodes 2–5.

Figure 12a illustrates the average PDR for each node in a low traffic load situation (1 pkt/minute). We can observe that both schedulers have similar performance, which is normal considering that the new implementation behaves like RBS Orchestra when the traffic is low enough to avoid congestion at the root’s children. The placement of nodes used for running the simulations results in a multi-hop topology. We expect to see the highest PDR coupled with the lowest delay for the root’s children. In the placement of nodes we used to run the simulations (Figure 8b), nodes 2–5 are the children of the root and have the highest rank among sender nodes. In Figure 12b, one can see that, in case of RBS Orchestra, the lowest PDR is for the root’s children, this being a sign of congestion. In contrast, the new scheduler behaves as we expect in high traffic situations.

**Figure 13:**
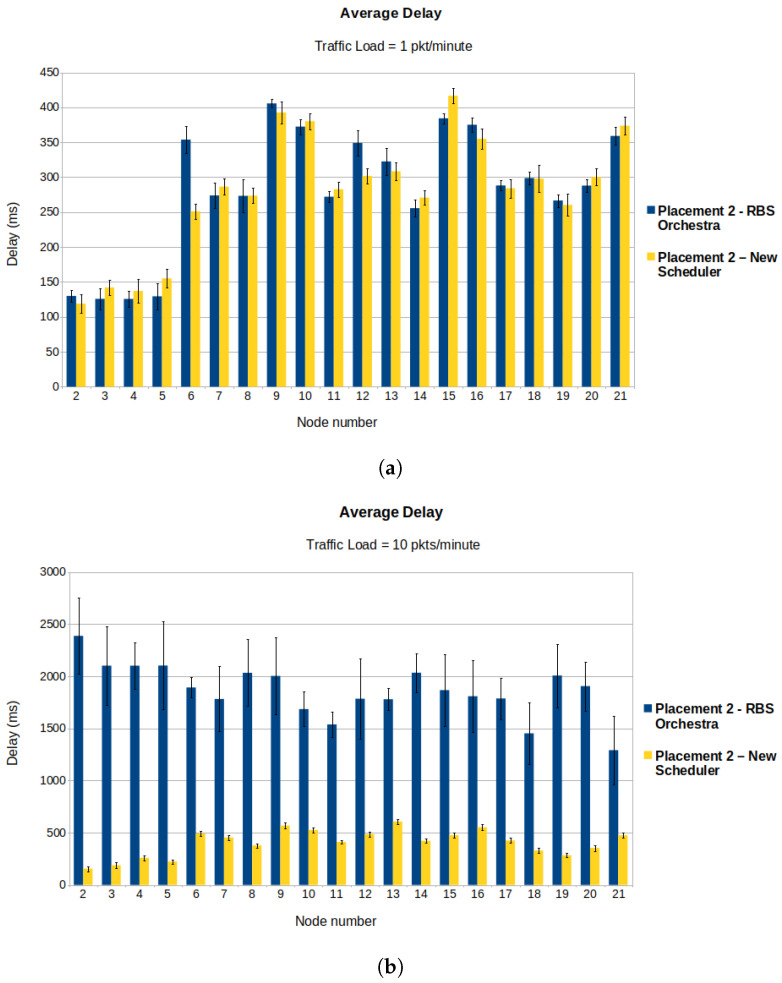
Average delay for each sender node in low and high traffic situations. (**a**) We observe that in normal traffic situation, the average delay is comparable for the two schedulers. (**b**) We observe that in high traffic situation for RBS Orchestra the average delay is highest for the root’s children, being nodes 2–5.

Figure 13a shows the average delay when the traffic is low. One can observe that nodes 2–5, the root’s children, for both schedulers, have the lowest delay. For RBS Orchestra, this is not true anymore when there is an increase in traffic and nodes become congested (Figure 13b). In this situation, the delay for the root’s children becomes comparable to or higher than the delay for the rest of the nodes.

#### 6.1.4. Average Radio Duty Cycle

**Figure 14:**
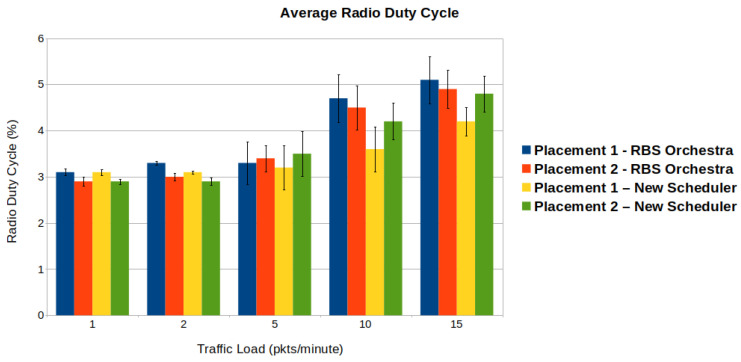
We observe that when it comes to average radio duty cycle, the new scheduler performs better than Orchestra in a high-traffic situation.

The average radio duty cycle represents the amount of time that a node has its radio on relative to the time of the simulation runs. The time that a node has its radio on is an indicator of power consumption, since the radio unit is one of the most energy-consuming components. Figure 14 shows the radio duty cycle in function of the traffic load. The radio duty cycle is comparable for both schedulers when the traffic is below 10 packets per minute. The increase observed for our scheduler is due to the additional cells allocated to accommodate the increase in traffic. For RBS Orchestra, the significant increase is related to contention leading to unstable links, multiple retransmissions, and additional control traffic. Analyzing the performance of the new scheduler, one can see that the radio duty cycle is higher for placement 2. This is explained by the placement of nodes and the resulting topology. On the one hand, the sink node has more children than in placement 1, and on the other hand, the nodes are disposed around the root and the root’s children, leading to higher chances of allocating more additional cells for all the children.

#### 6.1.5. Stability of the Network

**Figure 15:**
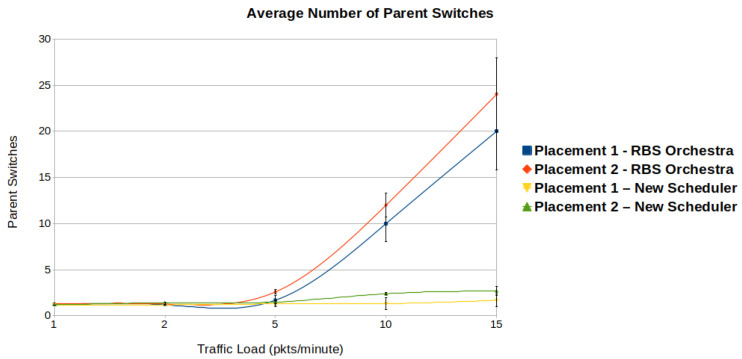
We observe that when it comes to stability, the new scheduler performs better than Orchestra, in high traffic situations.

We asses the stability of the network by looking at the number of parent switches. Figure 15 illustrates the number of parent switches in function of traffic load. One can observe that, when the traffic increases above 5 packets per minute, in case of RBS Orchestra, both topologies become unstable. For our new scheduler, the increase in number of parent switches is less significant, and remains below 3, regardless of the traffic load or topology. For both schedulers, the average number of parent switches is higher for placement 2. In placement 2, the nodes have more direct neighbors, which leads to higher interference and link instability, directly related to the number of parent switches.

In our simulations, RPL is in storing mode, which means that each parent switch represents a DIO-DAO message exchange. Considering this, the number of parent switches is correlated with the overhead. The overhead created by the control traffic is significantly higher in the case of RBS Orchestra.

### 6.2. Impact of Number of Nodes—Comparison with OSCAR

**Figure 16:**
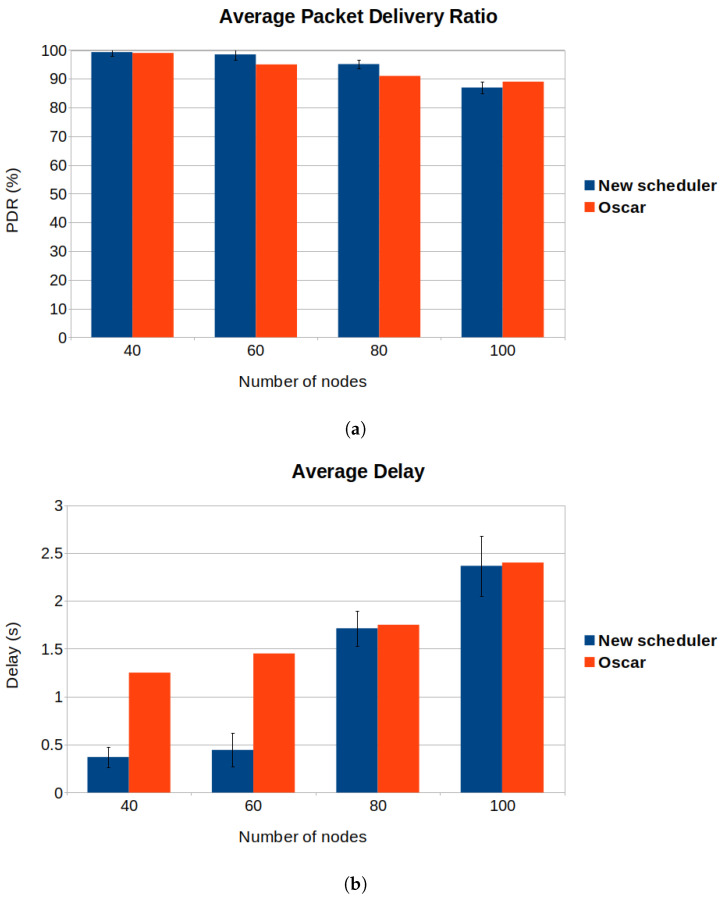

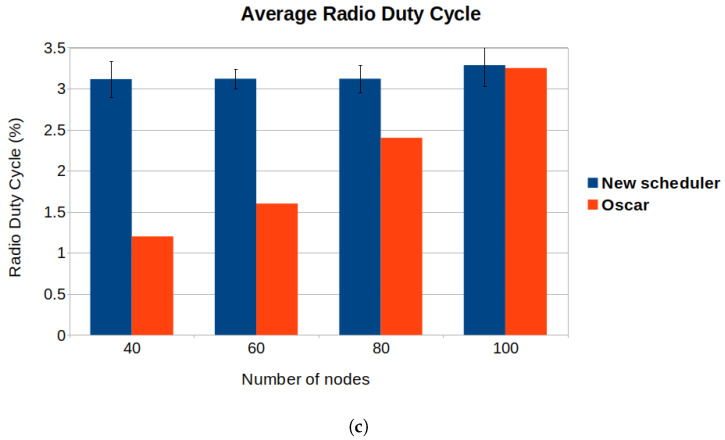
Comparison between OSCAR and the new scheduler considering different topology sizes. (**a**) We observe that the new scheduler has a higher PDR when the number of nodes is less than 100. (**b**) We observe that the new scheduler has a lower delay when the number of nodes is less than 80. (**c**) We observe that the new scheduler has a higher radio duty cycle.

We compare our implementation with OSCAR, an adaptive approach described in Section 3.2. Similar to OSCAR, the design principles of our scheduler are based on the idea that the nodes that suffer the most in convergecast traffic are the children of the root. When there is little traffic, OSCAR and our scheduler behave like RBS Orchestra. In OSCAR, the number of additional allocated cells is correlated to the rank of the node, in our implementation it is correlated with the subtree size, which makes our solution more suitable for unbalanced networks. Moreover, we only adapt the scheduler for the root and its children. To analyze the differences of the two schedulers, we study the impact of the number of nodes on the average delay, average PDR and average radio duty cycle (Figure 16).

We consider the same performance parameters as the authors of OSCAR. The desired number of nodes are placed on a 200 m × 200 m grid. The sender nodes are placed uniformly around the root node. We vary the number of sender nodes from 20 to 100 nodes. Each sender in the network transmits 2000 packets to the root. The traffic load is 1 pkt/minute, same as in [13]. To complete our figures and compare the schedulers, we took the values from the article that describes OSCAR, in Figure 9a–c, Section 5.3.2 of [13]. This also means we were restricted by the placement of nodes the authors used for analyzing the performance of OSCAR, and we could not compare the schedulers in unbalanced networks.

The authors of OSCAR use the rank of the node to attribute resources to the nodes. In OSCAR, each root’s child is allocated 1 cell every 6 cells. We consider the subtree size of the root’s children when allocating resources, thus in our case, the number of allocated cells can vary depending on the subtree size. We allocate 0.7, 1.06 or 1.4 cells every 6 cell. Considering the high number of nodes, we suspect each child of the root is allocated the maximum number of cells, 1.4 cells every 6 cells. We expect our scheduler to increase the average PDR, decrease the delay and increase the radio duty cycle when compared to OSCAR.

As expected, we see a 70% decrease in average delay and a slightly higher PDR for our scheduler, if the number of nodes in the network is less or equal to 80 (Figure 16a,b). This happens because, on one hand, we allocate more transmission opportunities for the root’s children than they do in OSCAR. On the other hand, we only adapt the scheduler of the root and the root’s children to tackle congestion. If the network has a high number of nodes, the performance of our scheduler will start decreasing because the congestion will become significant also for nodes farther from the root (Figure 16a).

In Figure 16c, one can see that the new scheduler has a higher average duty cycle, regardless of the number of nodes in the network. This can be explained by the fact that we allocate more transmission opportunities for the root’s children.

## 7. Conclusions

This article proposes a new scheduler for TSCH-based networks, a scheduler that is built on the principles of RBS Orchestra and adapts to traffic. In the case of high convergecast traffic, the most constrained nodes are the children of the root. In our approach, these nodes detect possible congestion by assessing the remaining number of packets in the queue and additional resources are allocated for the root and its children if they are needed. The subtree size is used to determine the number of additional cells that should be allocated for each child of the root. The subtree of a node contains all its descendants and can be obtained if RPL is in storing mode. It is important to consider the subtree size to avoid unfairness between the root’s children in case the network topology is unbalanced.

The new scheduler is evaluated in the Cooja simulator on two placement of nodes, each with one sink node and 20 sender nodes. The traffic load is varied to observe the adaptability of the scheduler. From the experiments, we observe that our scheduler makes RBS Orchestra adaptive to sudden traffic bursts: data transmission remains reliable, the delay is decreased, and the stability of the network is preserved. Allocating additional resources leads, of course, to an increase in radio duty cycle.

The new scheduler is also compared with OSCAR, a scheduler that also uses an adaptive approach. In this context, the impact of the number of nodes on the scheduler’s performance is also studied. Compared to OSCAR, the new scheduler slightly increases the PDR and decreases the delay with 70%, if the number of nodes in the network remains under a certain threshold, such that the congestion does not propagate farther than the root’s children. The downside is an increase in radio duty cycle. When the number of nodes in the network is above a particular value, OSCAR proves to be the superior implementation because it adapts the scheduler for all the nodes in the network.

Modifying the thresholds of our scheduler will allow to tune the approach to what is most important for the application, a decrease in latency and an increased PDR or longer lifetime. Future work should further analyze the thresholds for activating and deactivating the scheduler, and the number of needed cells. In case the traffic drops, the deactivation of the scheduler relies on the number of packets in the queue. It could be better to evaluate the use of the available cells instead of the remaining number of packets in the queue. Other traffic patterns can also be considered to further evaluate the proposed scheduler. It is also important to study the behavior of the new implementation on testbeds.

## Figures and Tables

**Table 1:** Strong points and limitations of discussed schedulers.

Scheduler	Approach	Strong Points	Limitations
**ALICE [7]**	Static link based	Separation between upstream and downstream traffic; scheduling of cells changes in time; early drop of packets	Not adaptive, allocates the same amount of cells regardless of traffic load
**Escalator [8]**	Static node based	cells allocated based on the subgraph of a node; increase in average duty cycle even for low traffic	Not adaptive and downward traffic is not supported; duty cycle is directly linked to the size of the routing table
**PAAS [9]**	Adaptive based on SBS Orchestra	Reduces the number of Rx cells if traffic load is low	Tx cells are allocated before Rx cells which might lead to packet loss; increase in overhead by extending the size of the DIO message
**TESLA [10]**	Adaptive with 2 slotframes for unicast traffic	The size of the unicast slotframes is adjusted based on traffic load	Introduces overhead when nodes communicate the slotframe size
**ATAS [11]**	Adaptive and packet prioritization	cells are allocated based on the number of remaining packets in the queue; priority packets are added at the head of the queue	The additional cells are allocated sequentially which might increase collision probability
**A3 [12]**	Adaptive and fully autonomous	Sender and receiver allocate cells independently based on traffic load; sender predicts traffic based on remaining packets in the queue; receiver estimates incoming traffic by sampling the channel	Increase in CPU overhead because of the complexity of the algorithm that estimates the number of additional cells
**OSCAR [13]**	Adaptive	The rank of the node is used to allocate additional cells in case of high traffic	Might allocate not needed cells in case of unbalanced networks
**MSF [14]**	Adaptive and distributed	Autonomous cells ensure minimal bidirectional communication; in case of traffic burst, additional cells are allocated through negotiation	Distributed scheduler which might lead to increased complexity in implementation

**Table 2:** Values of the thresholds used for exemplifying the allocation of additional time slots.

Threshold	Value
subtree_lower_threshold	3
subtree_upper_threshold	5
M	2
N	3

**Table 3:** Values for the parameters of the evaluation environment.

**Cooja Simulator**	Radio medium	UDGM: Distance Loss
	Random Seed	New random seed on reload
	Mote Type	Cooja mote
	Grid Dimensions	20 × 20 (200 m × 200 m)
	Transmission Range	50 m
	Interference Range	50 m
**Operating System**	Contiki-NG	Version 4.7
**RPL**	Mode of Operation	Storing mode
**Orchestra**	EB slotframe	397 slots
	Broadcast slotframe	31 slots
	Unicast slotframe	17 slots
	Mode	Receiver-based Shared
**New Scheduler**	EB slotframe	397 slots
	Broadcast slotframe	31 slots
	Unicast slotframe	17 slots
	activation_threshold	5
	deactivation_threshold	2
	subtree_lower_threshold	3
	subtree_upper_threshold	5
	M	2
	N	3
**Traffic**	Normal load	1 and 2 packets/minute
	Burst	5, 10 and 15 packets/minute
	# Packets sent by each node	1000: 250 normal traffic + 500 burst traffic
		+ 250 normal traffic
	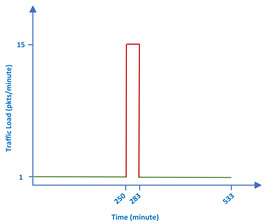	

## Data Availability

The data that support the findings of this study are available from the corresponding author, K.S., upon reasonable request.

## Abbreviations

The following abbreviations are used in this manuscript:

6TiSCH	IPv6 over the TSCH mode of IEEE 802.15.4e
ASN	Absolute Slotframe Number
CS	Common Shared
CSMA	Carrier Sense Multiple Access
DAO	Destination Advertisement Object
DIO	DODAG Information Object
DIS	DODAG Information Solicitation
DODAG	Destination Oriented Directed Acyclic Graph
EB	Enhanced Beacon
IANA	Internet Assigned Numbers Authority
IEEE-SA	Institute of Electrical and Electronics Engineering Standards Association
IEFT	Internet Engineering Task Force
IoT	Internet of Things
IPv6	Internet Protocol version 6
MAC	Medium Access Control
OSCAR	Optimized Scheduling Cell Allocation Algorithm for Convergecast
PDR	Packet Delivery Ratio
QoS	Quality of Service
RBS	Receiver-based Shared
RPL	IPv6 Routing Protocol for Low-Power and Lossy Networks
SBD	Sender-based Dedicated
SBS	Sender-based Shared
TDMA	Time Division Multiple Access
TSCH	Time-Slotted Channel Hopping
UDGM	Unit Disk Graph Medium
WSN	Wireless Sensor Network

## References

[1] Kurunathan H., Severino R., Koubaa A., Tovar E. (2018). IEEE 802.15.4e in a Nutshell: Survey and Performance Evaluation. IEEE Commun. Surv. Tutorials.

[2] Duquennoy S., Al Nahas B., Landsiedel O., Watteyne T. (2015). Orchestra: Robust Mesh Networks Through Autonomously Scheduled TSCH. Proceedings of the 13th ACM Conference on Embedded Networked Sensor Systems.

[3] Osterlind F., Dunkels A., Eriksson J., Finne N., Voigt T. Cross-Level Sensor Network Simulation with COOJA. Proceedings of the 31st IEEE Conference on Local Computer Networks.

[4] Alexander R., Brandt A., Vasseur J.P., Hui J., Pister K., Thubert P., Levis P., Struik R., Kelsey R., Winter T. (2012). RPL: IPv6 Routing Protocol for Low-Power and Lossy Networks.

[5] Levis P., Clausen T.H., Gnawali O., Hui J., Ko J. (2011). The Trickle Algorithm.

[6] (2012). IEEE Standard for Local and metropolitan area networks–Part 15.4: Low-Rate Wireless Personal Area Networks (LR-WPANs) Amendment 1: MAC sublayer.

[7] Kim S., Kim H.S., Kim C. (2019). ALICE: Autonomous link-based cell scheduling for TSCH. Proceedings of the 18th International Conference on Information Processing in Sensor Networks.

[8] Oh S., Hwang D., Kim K.H., Kim K. (2018). Escalator: An Autonomous Scheduling Scheme for Convergecast in TSCH. Sensors.

[9] Jung J., Kim D., Hong J., Kang J., Yi Y. Parameterized slot scheduling for adaptive and autonomous TSCH networks. Proceedings of the IEEE INFOCOM 2018—IEEE Conference on Computer Communications Workshops (INFOCOM WKSHPS).

[10] Jeong S., Paek J., Kim H.S., Bahk S. (2019). TESLA: Traffic-Aware Elastic Slotframe Adjustment in TSCH Networks. IEEE Access.

[11] Rekik S., Baccour N., Jmaiel M., Drira K., Grieco L.A. (2018). Autonomous and traffic-aware scheduling for TSCH networks. Comput. Netw..

[12] Kim S., Kim H.S., Kim C.k. (2021). A3: Adaptive Autonomous Allocation of TSCH Slots. Proceedings of the 20th International Conference on Information Processing in Sensor Networks (co-located with CPS-IoT Week 2021).

[13] Osman M., Nabki F. (2021). OSCAR: An Optimized Scheduling Cell Allocation Algorithm for Convergecast in IEEE 802.15.4e TSCH Networks. Sensors.

[14] Chang T., Vučinić M., Vilajosana X., Duquennoy S., Dujovne D.R. (2021). 6TiSCH Minimal Scheduling Function (MSF).

[15] Wang Q., Vilajosana X., Watteyne T. (2018). 6TiSCH Operation Sublayer (6top) Protocol (6P).

[16] Hou J., Jadhav R., Luo Z. (2017). Optimization of Parent-Node Selection in RPL-Based Network.

[17] Contiki-NG: The OS for Next Generation IoT Devices, 2022. original-date: 2017-05-13T17:37:59Z. https://github.com/contiki-ng/contiki-ng.

[18] Alvi S.A., Hassan F.u., Mian A.N. On the energy efficiency and stability of RPL routing protocol. Proceedings of the 2017 13th International Wireless Communications and Mobile Computing Conference (IWCMC).

